# The Disturbance of the Antioxidant System Results in Internal Blue Discoloration of Postharvest Cherry Radish (*Raphanus sativus* L. var. *radculus pers*) Roots

**DOI:** 10.3390/foods12030677

**Published:** 2023-02-03

**Authors:** Xingyu Wang, Yu Liu, Wenting Zhao, Pan Wang, Shuang Zhao, Xiaoyan Zhao, Dan Wang

**Affiliations:** 1 Beijing Key Laboratory of Fruits and Vegetables Preservation and Processing, Key Laboratory of Vegetable Postharvest Processing, Ministry of Agriculture and Rural Affairs, Institute of Agri-Food Processing and Nutrition, Beijing Academy of Agriculture and Forestry Sciences, Beijing 100097, China; 2 College of Food Science and Engineering, Beijing University of Agriculture, Beijing 102206, China

**Keywords:** cherry radish, blue discoloration, antioxidant system, ROS, 4-hydroxyglucobrassicin

## Abstract

Internal blue discoloration in cherry radish (*Raphanus sativus* L. var. *radculus pers*) roots can appear after harvest. The antioxidant system and content of reactive oxygen species (ROS) will affect the blue discoloration. Currently, the reason for the blue discoloration is not yet clear. In order to reveal the mechanism of the blue discoloration of cherry radish, we selected the blue discolored cherry radish as the research object and the white cherry radish as the control. The difference in the antioxidant system between them were compared, including related enzymes and non-enzymatic antioxidants in this system. Meanwhile, the changes in the contents of 4-hydroxyglucobrassicin as a precursor substance and ROS were compared. The results showed that the activities of typical antioxidant enzymes decreased and the cycle of Glutathione peroxidase (GPX) and Ascorbic acid–Glutathione (ASA–GSH) was disturbed, leading to the reduction of antioxidant effect and the failure of timely and effective decomposition of superoxide anions (O_2_^•−^) and hydrogen peroxide (H_2_O_2_). In addition, the elevated level of O_2_^•−^ and H_2_O_2_ led to the disorder of the antioxidant system, while the 4-hydroxybrassinoside was oxidized under the catalysis of peroxidase (POD) and eventually led to the internal blue discoloration in cherry radish. These results can provide a theoretical basis for solving the blue discoloration problem.

## 1. Introduction

Radish is a vegetable species of brassica vegetables planted all over the world, and its main edible part of the plant is the thickened main root [1]. Additionally, it has numerous varieties which differ in size, color and cultivation requirements [2]. In the previous studies, internal blue discoloration of roots from 20 to 150 mm during postharvest storage at approximately 20 °C [3] was observed in Daikon, the Japanese white radish (*Raphanus sativus*). This physiological phenomenon is related to oxidative stress in radish roots [4]. The 4-hydroxyglucobrassicin, as the precursor, is oxidized by the H_2_O_2_ or other ROS in the presence of POD, leading to the discoloration [5,6,7]. The high content of oxidation substances promotes the discoloration of the radish root. Cherry radish (*Raphanus sativus* L. *var. radculus pers*) is a type of four-season radish with a bright color which can be used in salad dressings [8]. In our laboratory, we have found that cherry radish also appears to have blue discoloration during postharvest storage, and this discoloration occurs in the whole fleshy root of the cherry radish.

ROS is the typical oxidizing substance in plants, which is produced by various metabolic pathways and plays an important role in the physical process [9,10]. In normal conditions, the metabolism of ROS is balanced [11]. It is known that the overproduction of ROS can lead to the browning of harvested longan fruit [12] and the yellowing of postharvest broccoli [11]. H_2_O_2_ and O_2_^•−^ are the two common forms of ROS, and their levels can be adjusted by the antioxidant systems [13]. An antioxidant system exists in plants, including antioxidant enzymes and non-enzyme antioxidants, which can eliminate the overproduction of ROS and maintain the ROS homeostasis [14,15]. The presence of a plant antioxidant system is particularly important to regulate and balance ROS through antioxidant enzymes, the GPX cycle and the ASA–GSH cycle. POD and polyphenol oxidase (PPO) are typical oxidoreductases in plants, which can participate in the regulation of ROS. Moreover, studies have demonstrated that the POD and PPO play a major role in the discoloration of fruits during postharvest storage, such as longan, litchi, rambutan and banana [16,17,18,19]. How the antioxidant system is regulated in cherry radish has not been reported.

In order to investigate the mechanism of blue discoloration in the cherry radish, we selected the blue discolored cherry radish and the white cherry radish as the materials of research in this study. Then, we explored the effect of internal blue discoloration on the cherry radish, including color, precursor, ROS and antioxidant system, with the aim of elucidating the possible mechanisms involved. The results would provide a theoretical basis for the inhibition of cherry radish internal bluing during postharvest storage.

## 2. Results and Discussion

### 2.1. Appearance and Color

**Figure 1:**
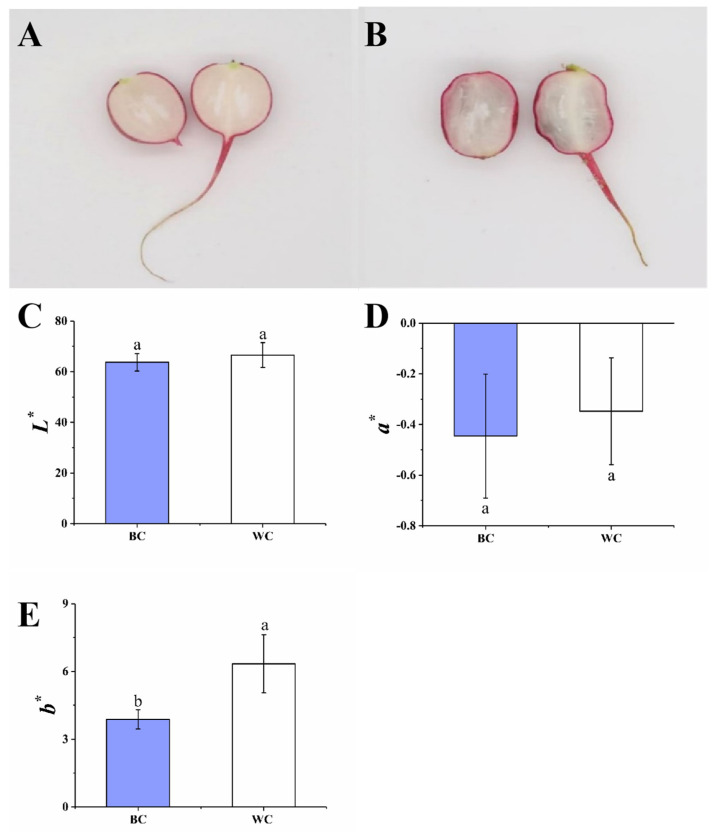
Cross-section of cherry radishes and change in L*, a* and b* values after three days of storage. The uncolored cherry radishes (**A**); the blue discolored of cherry radishes (**B**); L* value of two groups of cherry radish (**C**); a* value of two groups of cherry radish (**D**); b* value of two groups of cherry radish (**E**). BC = blue discolored cherry radishes, WC = uncolored cherry radishes. Each value in figures was presented as the mean ± standard error (*n* = 3). Different letters represent the statistically significant differences between the different treatment groups (*p* < 0.05).

As illustrated in Figure 1A,B, the pictures show the overall visual quality of cherry radishes stored for three days. Uncolored cherry radishes were white in cross section and had no other variegated color (Figure 1A). The blue substance was found in the cross section of the cherry radishes (Figure 1B). To confirm the visual observations of blue discoloration, the L*, a*, and b* values (Figure 1C–E) were measured in this study. There was no significant change in L* and a* values between the two samples (*p* > 0.05). Meanwhile, compared with the white cherry radish, the b* value of blue discoloration in the cherry radish decreased from 6.34 to 3.87 (*p* < 0.05), which indicated a direct correlation with blue change. The two sets of cherry radishes mentioned previously would be used for subsequent analysis.

### 2.2. 4-Hydroxyglucobrassicin Content

**Figure 2:**
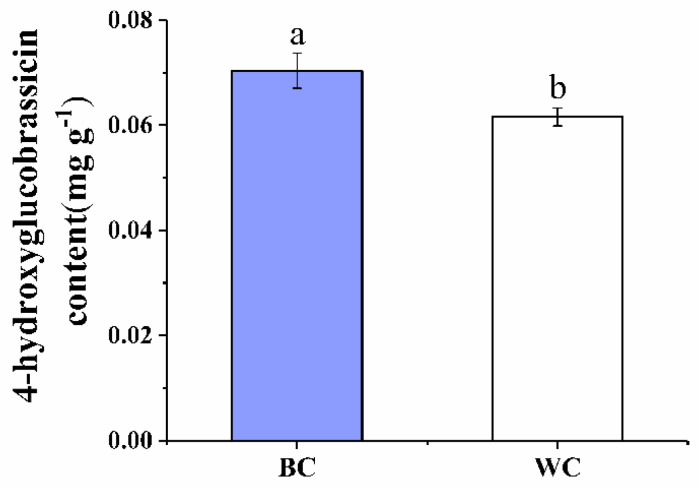
Effect of internal blue discoloration on the levels of 4-hydroxyglucobrassicin. BC = blue discolored cherry radishes, WC = uncolored cherry radishes. Each value in figures was presented as the mean ± standard error (*n* = 3). Different letters represent the statistically significant differences between the different treatment groups (*p* < 0.05).

As Figure 2 shows, the content of 4-hydroxyglucobrassicin had a significant difference between the discolored samples and samples without discoloration (*p* < 0.05); 0.070 mg g^−1^ and 0.062 mg g^−1^, respectively. Studies have shown that the content of 4-hydroxyglucobrassicin is consumed in the process of discoloration during the storage, apparently [3,20]. Compared with the sample without discoloration, the sample with blue discoloration had the higher content of 4-hydroxyglucobrassicin in the beginning, which provided the material for the production of blue discoloration in the cherry radish. Hence, it could be inferred that the 4-hydroxyglucobrassicin was also the precursor in the blue discoloration of the cherry radish. Additionally, the higher content of 4-hydroxyglucobrassicin was one of the reasons for the blue discoloration phenomenon.

### 2.3. H_2_O_2_ and O_2_^•−^ Content

**Figure 3:**
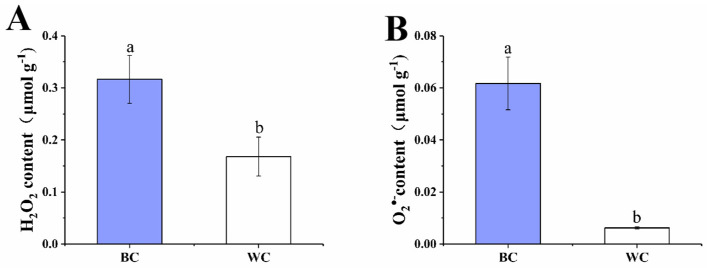
Effect of internal blue discoloration on the levels of H_2_O_2_ (**A**) and O_2_^•−^ (**B**). BC = blue discolored cherry radishes, WC = uncolored cherry radishes. Each value in figures was presented as the mean ± standard error (*n* = 3). Different letters represent the statistically significant differences between the different treatment groups (*p* < 0.05).

The ROS content is one of the indexes for evaluating the degree of oxidative stress [21]. In our study, H_2_O_2_ and O_2_^•−^ were selected for evaluating the degree of oxidative stress. As Figure 3A,B presented, the contents of O_2_^•−^ and H_2_O_2_ of cherry radish increased significantly (*p* < 0.05) after blue discoloration. The O_2_^•−^ content of blue discolored samples (0.062 μmol/g) was about 10 times that of samples without blue discoloration (0.006 μmol/g). The half-life of O_2_^•−^ is very short and it does not cause more cellular damage by itself; however, O_2_^•−^ easily transforms into more toxic forms of ROS [22]. The H_2_O_2_ content of the discolored samples was 0.32 μmol/g, which was twice that of the samples without discoloration (0.17 μmol/g). H_2_O_2_ is a by-product of aerobic metabolism, which could be synthesized by both enzymatic and nonenzymatic cellular processes such as photorespiration, redox reaction and electron chain [23]. Additionally, H_2_O_2_ is a moderately reactive and long-lived molecule, it plays an important role in various physiological processes, but the overproduction could cause oxidative damages of the cell [24]. In our study, excessive O_2_^•−^ and H_2_O_2_ were observed in the discolored cherry radish, which indicated the occurrence of oxidative stress. This is consistent with the previous findings of elevated H_2_O_2_ and O_2_^•−^ content in blue discolored radishes [7]. Moreover, mechanisms of blue discolored radish occurrence were related to oxidative stress [5].

### 2.4. The Antioxidant System

#### 2.4.1. SOD and CAT Activity

**Figure 4:**
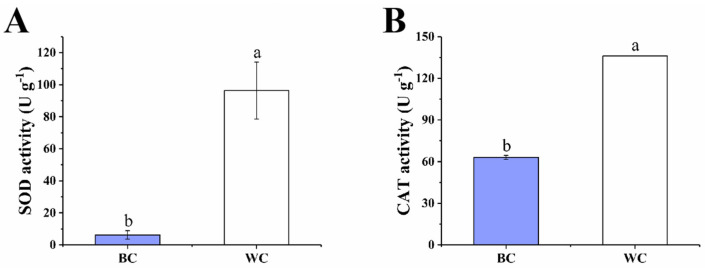
Effect of internal blue discoloration on the SOD activity (**A**) and CAT activity (**B**). BC = blue discolored cherry radishes, WC = uncolored cherry radishes. Each value in figures was presented as the mean ± standard error (*n* = 3). Different letters represent the statistically significant differences between the different treatment groups (*p* < 0.05).

The results showed that the activity of the antioxidant enzymes of the blue discolored cherry radish were significantly lower than the uncolored cherry radish (*p* < 0.05), especially superoxide dismutase (SOD) and catalase (CAT) (Figure 4A,B). The activity of SOD was 6.26 U g^−1^ in the blue discolored cherry radish and 96.37 U g^−1^ in the uncolored cherry radish. SOD is one of the metalloenzymes present in all aerobic cells, which is the first line of defense against O_2_^•−^, and it can catalyze the decomposition of O_2_^•−^ into H_2_O_2_. The low activity of SOD in the blue discolored cherry radish reduced the conversion of O_2_^•−^ to H_2_O_2_, so that O_2_^•−^ could not be decomposed and aggregated through this transformation. The CAT is a kind of product of the peroxidase enzyme, containing hemoglobin, which exists in the chloroplast, mitochondria and cytoplasm catalytic decomposition of H_2_O_2_ molecules to H_2_O and O_2_ [25]. The CAT activity of the white cherry radish was 136.08 U g^−1^, which was significantly higher than the blue discolored cherry radish (63.08 U g^−1^). The removal of O_2_^•−^ is transformed into H_2_O_2_ by SOD, and then decomposed into H_2_O and O_2_ by CAT.

Both enzymes showed reduced activity in the blue discolored samples. The reduction in CAT activity also reduced the degree of decomposition of H_2_O_2_ into H_2_O, resulting in a partial aggregation of H_2_O_2_. The decreased activity of the two enzymes explained the change in ROS content, which was consistent with the results of the H_2_O_2_ and O_2_^•−^ content measurements. In radish roots, SOD can suppress ROS [5]. As reported by Zhang et al., the gene expression of SOD and GPX was upregulated in the study, which may be related to differences in the varieties of radish itself [7].

#### 2.4.2. The GPX Cycle

**Figure 5:**
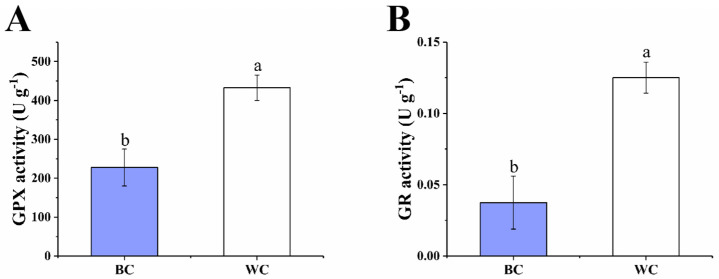
Effect of internal blue discoloration on the GPX activity (**A**), GR activity (**B**). BC = blue discolored cherry radishes, WC = uncolored cherry radishes. Each value in figures was presented as the mean ± standard error (*n* = 3). Different letters represent the statistically significant differences between the different treatment groups (*p* < 0.05).

The GPX cycle is an important way for plants to remove ROS, which is present in all living organisms and can reduce H_2_O_2_ to produce H_2_O [26]. As presented in Figure 5A, the GPX activity in blue discolored cherry radish was 227.50 U g^−1^ and the white cherry radish was 432.68 U g^−1^; the difference in activity was nearly two-fold. With the decrease of GPX activity, H_2_O_2_ cannot decompose into water in time and its content increases. Furthermore, GPX use GSH as a cofactor, and hence convert to glutathione disulfide (GSSG) [17]. Glutathione reductase (GR) acts as an antioxidant enzyme and catalyzes the generation of GSH from GSSG using the triphosphopyridine nucleotide (NADPH) as the sole reducing power and electron donor [27]. The GR activities of blue discolored cherry radish and white cherry radish were 0.03 U g^−1^ and 0.12 U g^−1^, respectively (Figure 5B). At the same time, the decreased GR activity blocked the conversion of GSSG, leading to a decrease in GSH content. In the GPX cycle, the activity of antioxidant enzymes and the content of GSH decreased, resulting in the failure of H_2_O_2_ decomposition and aggregation in time, so it could not effectively play the antioxidant role. It can be deduced from the result that the antioxidant enzymes in the blue discolored cherry radish were inhibited, resulting in the overproduction of ROS and oxidative stress, which may cause the internal blue discoloration of the cherry radish during postharvest storage. Similarly, reduced gene expression levels of GPX and GSH were revealed in the blue discolored radish roots [7].

#### 2.4.3. ASA–GSH Cycle

**Figure 6:**
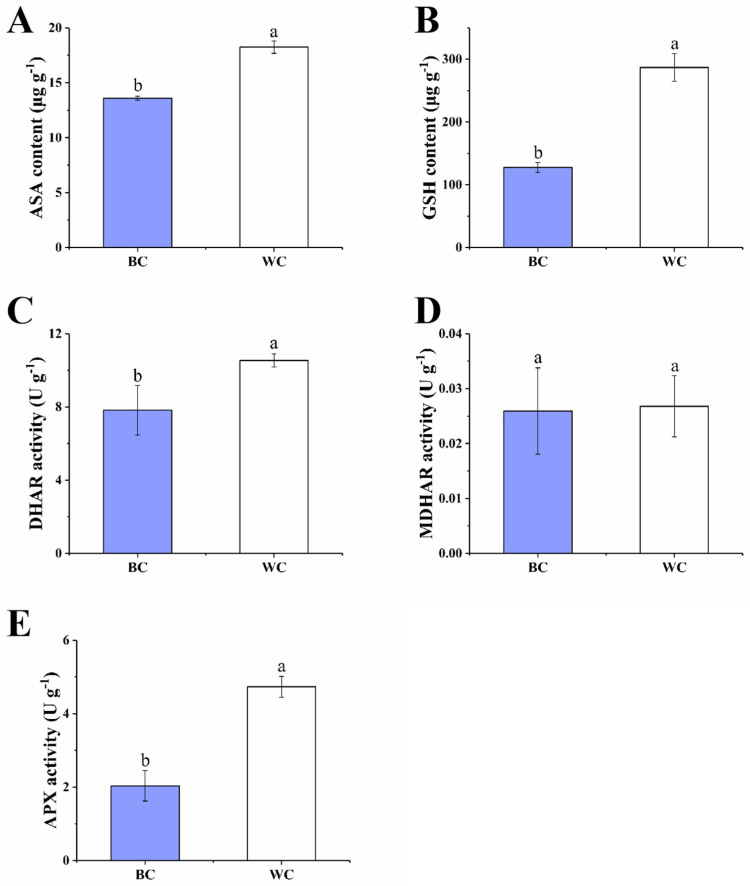
Effect of internal blue discoloration on the ASA–GSH cycle. ASA content (**A**), GSH content (**B**), DHAR activity (**C**), MDHAR activity (**D**), APX activity (**E**). BC = blue discolored cherry radishes, WC = uncolored cherry radishes. Each value in the figures was presented as the mean ± standard error (*n* = 3). Different letters represent the statistically significant differences between the different treatment groups (*p* < 0.05).

The ASA–GSH cycle also plays a key role in the antioxidant system, which can balance the oxidative and reductive environment [28]. As shown in Figure 6A,B, the contents of Asa ascorbic acid (ASA) and GSH were significantly different in two types of cherry radish (*p* < 0.05). The ASA content was 13.59 mg 100g^−1^ in the blue discolored cherry radish and 18.24 mg 100 g^−1^ in the uncolored cherry radish. ASA is the most abundant antioxidant in plants, which is also one of the most powerful compounds for reducing ROS, by the ASA–GSH cycle or directly [29]. The decrease in ASA content also predicts a reduction in the ability to scavenge ROS. Additionally, the content of GSH in the blue discolored cherry radish and uncolored cherry radish were 127.51 μg g^−1^ and 286.82 μg g^−1^, respectively. As a reductant of ROS and a substrate for certain peroxidases, GSH plays a vital antioxidant role in plants. A higher content of GSH contributes to relieve drought, salinity, high/low temperatures and heavy metal [25]. This change in content would also lead to a decrease in its antioxidant effect, as in the case of ASA. Moreover, dehydroascorbate reductase (DHAR), monodehydroascorbate reductase (MDHAR), GR and ascorbate peroxidase (APX) also participate in the ASA–GSH cycle and play an important role. The key enzyme of the ASA–GSH cycle in the blue discolored cherry radish was lower than the uncolored cherry radish in various degrees. As shown in Figure 6C, the DHAR activity of the blue discolored cherry radish was 7.82 U g^−1^; it was significantly lower than the enzyme activity in the white cherry radish (10.53 U g^−1^). DHAR is a key enzyme regulating the oxidation reduction state of ASA, which can promote the reduction of docosahexaenoic acid (DHA) to regenerate ASA, which is used to scavenge free radicals in the body and protect plants from damage [30]. The MDHAR activities of blue discolored cherry radish and the white cherry radish were 0.026 U g^−1^ and 0.027 U g^−1^, respectively (Figure 6D), which indicated that the effects of MDHAR on the blue discoloration of cherry radish are small. MDHAR acts as an antioxidant and promotes the regeneration of ASA [31,32]. The APX activity of the white cherry radish was 4.73 U g^−1^, and the blue discolored cherry radish group was 2.03 U g^−1^ (*p* < 0.05) (Figure 6E). APX has the same role as CAT, but they are two H_2_O_2_-scavenging enzymes. APX might be responsible for the fine modulation of ROS for signaling, whereas CAT might be responsible for the removal of excess ROS during stress [25].

Research has also shown that the higher enzyme activities of the ASA–GSH cycle largely contribute to H_2_O_2_ elimination [33]. In the ASA–GSH cycle, the main substances involved include ASA, GSH, APX, DHAR and GR, with the exception of MDHAR, whose content and activity were decreased to different degrees. With the decrease of APX content, H_2_O_2_ could not be decomposed effectively and its content increased. The activity of DHAR and GR decreased by different degrees, and the ability of DHAR and GR to catalyze the generation of ASA and GSH decreased. Therefore, in the blue discolored cherry radish, the lower ASA and GSH contents and DHAR and GR activities increased ROS production and oxidative stress. This was consistent with the finding that blue discoloration of the radish root was associated with oxidation and reduction systems [7]. Specifically, the gene expression of ROS-related substances was upregulated, while genes for substances with antioxidant effects (ASA, GSH, GPX, APX) were downregulated.

### 2.5. POD and PPO Activity

**Figure 7:**
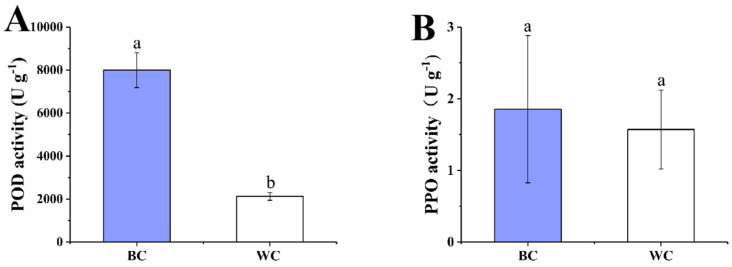
Effect of internal blue discoloration on the levels of POD activity (**A**) and PPO activity (**B**). BC = blue discolored cherry radishes, WC = uncolored cherry radishes. Each value in the figures was presented as the mean ± standard error (*n* = 3). Different letters represent the statistically significant differences between the different treatment groups (*p* < 0.05).

POD and PPO are typical oxidoreductases in plants which may cause the undesired change in color of fruits and vegetables during postharvest storage [34,35]. Therefore, it is inferred that the POD and PPO may play a vital role in the process of blue discoloration in the cherry radish during postharvest storage; thus, the activities of two enzymes were measured in our study. As shown in Figure 7A, the activity of POD was significantly higher in the blue discolored samples (7998.47 U g^−1^) than samples without blue discoloration (2122.31 U g^−1^). Teranishi et al. reported for the first time that 4-hydroxyglucobrasicin produces blue substances under the catalysis of POD, and Zhang et al. also found that POD acts as an oxidation factor to oxidize precursor substances to blue [3,7]. Therefore, it can be inferred that POD is also a key enzyme in the transformation process of 4-hydroxyglucobrassicin. However, Figure 7B showed that the PPO activity had almost the same level of two types of samples; there was no significant difference. The results showed that the POD, but not PPO, played the main role in the blue transformation of cherry radish.

### 2.6. Mechanism Discussion

**Figure 8:**
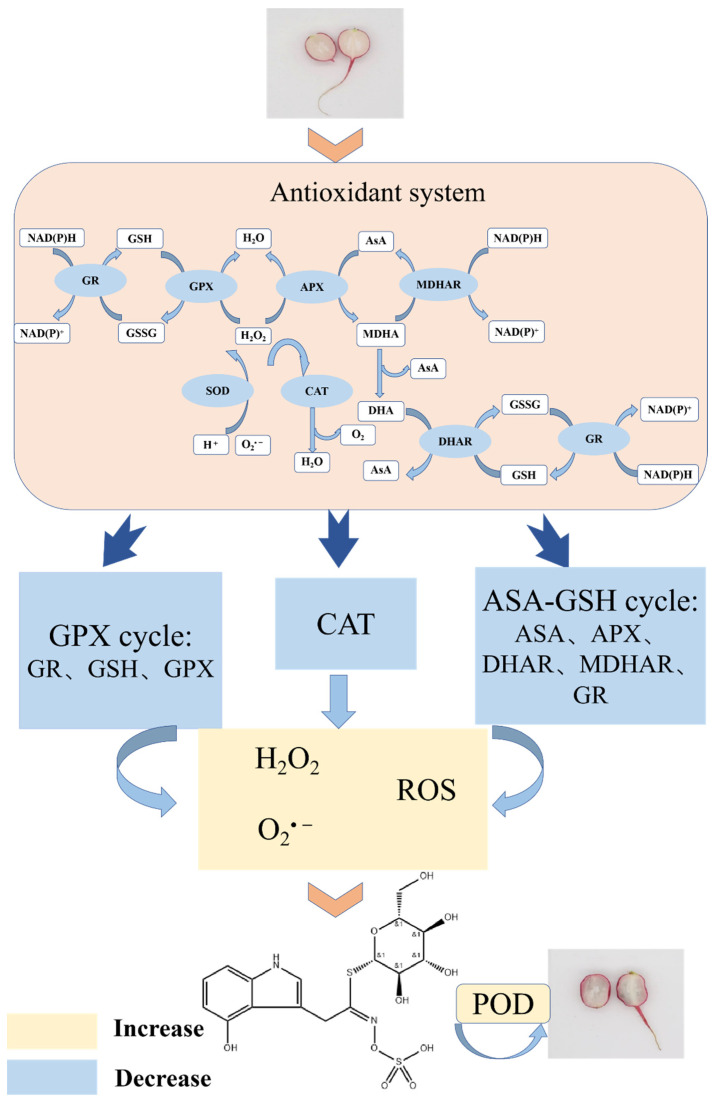
Proposed mechanism for the internal blue discoloration of postharvest cherry radish.

Researchers have reported that the blue discoloration of the radish root was related to oxidation and reduction systems [3,36]. By summarizing the above results and combining the relationship between antioxidant systems and ROS, we speculated the possible mechanism of cherry radish blue discoloration (Figure 8). This mechanism was closely related to the balance/disorder of substances and enzymes in the antioxidant system. Firstly, the removal of O_2_^•−^ was transformed into H_2_O_2_ by SOD, and then decomposed into H_2_O and O_2_ by CAT, the GPX cycle and the ASA–GSH cycle. In the GPX cycle, the GSH content, GR and GPX activities in blue discolored cherry radishes were lower than those in the white cherry radish group. The ability of GR to catalyze GSSG to generate GSH and GPX to decompose H_2_O_2_ decreased. There was also a phenomenon in the ASA–GSH cycle that the decomposition effect of H_2_O_2_ decreased and the activity of related enzymes to catalyze the production of ASA and GSH decreased. In the cycle of ASA–GSH, the enzymes involved in the regulation of blue discoloration were mainly DHAR and APX, but not MDHAR. Although ASA and GSH as reducing substances were thought to delay the discoloration of fruits and vegetables, their content was reduced and the production pathway was inhibited in the blue discolored cherry radish [37,38]. The up-regulation of oxidase gene expression and the down-regulation of reducing substance gene expression in blue-transformed radish were thought to be associated with blue transformation [7]. This was consistent with the findings in this study; that the content of reducing substances decreased while oxidase activity increased. The reduction in the content of major enzymes and antioxidants in the ROS scavenging pathway led to the aggregation of ROS without effective scavenging, which was accompanied by the disorder of the antioxidant system. At the same time, the activity of POD as an oxidizing factor was enhanced, which promoted the blue transformation of the precursor substance. The occurrence of the above conditions eventually led to the oxidation of 4-hydroxyglucobrasicin and the internal blue discoloration of the cherry radish.

## 3. Materials and Methods

### 3.1. Materials and Reagents

The cherry radishes (*Raphanus sativus* L. var. *radculus pers*) were purchased from a commercial market, Guoxiangsiyi (Beijing, China). The 4-hydroxyglucobrassicin was purchased from PhytoPlan (Heidelberg, Germany). The methanol was purchased from Thermo Fisher Scientific (China) Co., Ltd. (Shanghai, China). The trifluoroacetic acid (TFA) was purchased from Sigma-Aldrich (St. Louis, MO, USA). The ASA was purchased from Biotopped (Beijing, China). Additionally, the ammonium molybdate was purchased from AI Qaeda chemical plant (Tianjin, China). Other chemicals were obtained from Beijing Chemical Plant (Beijing, China).

### 3.2. Treatment

Samples of cherry radish were uniform in color and maturity, without disease, mechanical injury, or decay. The samples were stored at 28 °C, 45% relative humidity; after 3 days, a part of samples were discolored. The discolored samples and the samples without discoloration were selected for determination. After evaluating the color, the samples were immediately frozen in liquid nitrogen and ground using a mill grinder (A11 B S25, IKA, Germany). Then, they were stored at −80 °C for further analyses, including the precursor content, ROS content, enzymatic activities and non-enzymatic antioxidants content.

### 3.3. Color Measurements

The surface color of cherry radish was measured by a colorimeter CM-700d1 (Konica Minolta, Inc., Tokyo, Japan), and parameters were measured three times at three different points on each cherry radish and expressed as L* (L* = 0 corresponds to black; L* = 100 corresponds to white), a* (negative indicates green and positive indicates red), and b* (negative indicates blue and positive indicates yellow).

### 3.4. 4-Hydroxyglucobrassicin Determination

The determination of 4-hydroxyglucobrassicin was carried out by high-performance liquid chromatography (HPLC), and the method was performed as described by Teranishi and Masayasu [5]. Then, the samples (2 g) were extracted with 1 mL methanol in an ice bath and centrifuged at 8000 rpm for 5 min at 0 °C. The supernatant was filtered through a 0.22 μm membrane filter for future determination. The extract was used for HPLC analysis using a 1260 HPLC system (Agilent, Santa Clara, CA, USA) with a diode array detector (DAD) and an X Bridge C18 column (Waters, Ireland) (250 × 4.6 mm i.d., 5 µm) at 20 °C. The mobile phase was a mixture of aqueous 0.1% (*v*/*v*) trifluoroacetic acid (TFA) solution (A) and methanol solution (B). The flow rate was 0.8 mL/min in a linear gradient starting from 100% A and reaching 50% A in 20 min. The injection volume of the sample was 20 μL and the determination wavelength was 280 nm, using external standard as quantitative method. The standard curve was linear between 0.05 and 1 mg mL^−1^.

### 3.5. H_2_O_2_ and Superoxide Anion (O_2_^•−^) Measurements

The measurements of H_2_O_2_ and O_2_^•−^ were carried out using commercial kits (Solarbio, Beijing, China). For O_2_^•−^ measurement, samples (1 g) were added to 1 mL extraction solvent, then the mixture was ground in an ice bath and centrifuged at 12,000 rpm for 20 min at 4 °C. Additionally, for H_2_O_2_ measurement, samples (0.5 g) were extracted with 1 mL extraction solution in an ice bath, and after grinding the mixture was centrifuged at 8000 rpm for 10 min at 4 °C. The supernatant was used to analyze the contents of O_2_^•−^ and H_2_O_2_ according to the manufacturer’s instructions. The absorbance was measured at 530 nm and 415 nm by UV-1800 spectrophotometer (Shimadzu, Kyoto, Japan). The results are expressed in μmol g^−1^.

### 3.6. Determination of Enzyme Activities

The activities of POD, PPO, SOD, GPX, CAT, APX, MDHAR, DHAR and GR were measured by commercial kits (Solarbio, Beijing, China) and a UV-1800 spectrophotometer (Shimadzu, Kyoto, Japan). Firstly, samples were extracted by extraction solvent (solid–liquid ratio is 1:5), the mixture was ground in an ice bath, and centrifuged at 10,000 rpm for 10 min at 4 °C to obtained the enzyme extract. The next steps were measured according to the manufacturer’s instructions. The results are expressed in U g^−1^.

### 3.7. Determination of ASA and Reduced Glutathione (GSH) Content

The content of ASA was determined using a molybdenum blue colorimetric method with minor modification [39]. For ASA measurement, each sample (4 g) was extracted with 3 mL oxalic acid solution, and the extract (1.5 mL) after filtration was added to 0.5 mL metaphosphoric acid–acetic acid solution, 1 mL sulfuric acid solution (5%) and 2 mL ammonium molybdate solution. Then, the solution was diluted to 25 mL with distilled water. The absorbance was measured by UV-1800 spectrophotometer (Shimadzu, Kyoto, Japan) at 705 nm. The standard curve was linear between 0.025 and 0.8 mg ASA. The content of ASA was calculated with the following formula (1):

The content of ASA (mg × 100 g^−1^) = (C × V) ÷ (V_1_ × W) × 100 (1)

where C was the ASA mass calculated from the standard curve (mg), V was the volume of the extract (mL), V_1_ was the volume of the tested sample (mL) and W was the quality of the sample (g).

For GSH measurement, the samples were flushed twice by phosphate-buffered solution (PBS), firstly, then the samples were accurately quantified (0.2 g) and extracted with extraction solvent (1 mL). The mixture was centrifuged at 8000 rpm for 10min at 4 °C, and measured by a commercial kit (Solarbio, Beijing, China) according to the manufacturer’s instruction. In addition, the absorbance was measured by UV-1800 spectrophotometer (Shimadzu, Kyoto, Japan) at 412 nm. The results are expressed in μg g^−1^. The standard curve was made by 3.125–100 μg mL^−1^ GSH.

### 3.8. Statistical Analysis

All measurements were repeated three biological times, and each value in figures was presented as the mean ± standard error. All the data were analyzed with independent samples *t*-tests of comparison of means using SPSS Statistic 25 (IBM, Armonk, NY, USA). The value of *p* < 0.05 represents statistical significance. The different letter above the bars represented significant difference (*p* < 0.05).

## 4. Conclusions

Blue discoloration will occur in cherry radishes during post-harvest storage. Compared with the uncolored cherry radish, the blue discolored cherry radish had the higher content of ROS. The higher content of 4-hydroxyglucobrassicin as the precursor was oxidized by the overproduction of ROS due to the disturbance of the antioxidant system under the catalysis of POD, and blue discoloration in cherry radish appeared. This study provides a theoretic basis and experiment foundation for the inhibition of internal blue discoloration of the cherry radish during postharvest storage.

## Data Availability

All data are included in the main text.

## Abbreviations

ROS	Reactive oxygen species
GPX	Glutathione peroxidase
GSH	Glutathione
ASA–GSH	Ascorbic acid–Glutathione
O_2_^•−^	Superoxide anions
H_2_O_2_	Hydrogen peroxide
POD	Peroxidase
PPO	Polyphenol oxidase
SOD	Superoxide dismutase
CAT	Catalase
GSSG	Glutathione disulfide
GR	Glutathione reductase
NADPH	Triphosphopyridine nucleotide
DHAR	Dehydroascorbate reductase
MDHAR	Monodehydroascorbate reductase
APX	Ascorbate peroxidase
DHA	Docosahexaenoic acid

## References

[1] Xie Y., Ying J., Tang M., Wang Y., Xu L., Liu M., Liu L. (2021). Genome–wide identification of AUX/IAA in radish and functional characterization of RsIAA33 gene during taproot thickening. Gene.

[2] Zhang X., Liu T., Wang J., Wang P., Qiu Y., Zhao W., Pang S., Li X., Wang H., Song J. (2021). Pan-genome of Raphanus highlights genetic variation and introgression among domesticated, wild, and weedy radishes. Mol. Plant.

[3] Teranishi K., Masayasu N. (2016). Structure of a Precursor to the Blue Components Produced in the Blue Discoloration in Japanese Radish (*Raphanus sativus*) Roots. J. Nat. Prod..

[4] Lee J.G., Lim S., Kim J., Lee E.J. (2017). The mechanism of deterioration of the glucosinolate-myrosynase system in radish roots during cold storage after harvest. Food Chem..

[5] Teranishi K., Masayasu N., Masuda D. (2016). Mechanism Underlying the Onset of Internal Blue Discoloration in Japanese Radish (*Raphanus sativus*) Roots. J. Agric. Food Chem..

[6] Teranishi K., Nagata M. (2018). Prediction and suppression of internal blue discoloration in roots of daikon, the Japanese radish (*Raphanus sativus* L.). Food Sci. Nutr..

[7] Zhang Y., Zhao X., Ma Y., Zhang L., Jiang Y., Liang H., Wang D. (2021). Transcriptome and metabolome profiling to elucidate mechanisms underlying the blue discoloration of radish roots during storage. Food Chem..

[8] Guo R., Li W., Wang X., Chen B., Huang Z., Liu T., Chen X., XuHan X., Lai Z. (2019). Effect of photoperiod on the formation of cherry radish root. Sci. Hortic..

[9] Foyer C.H. (2020). Making sense of hydrogen peroxide signals. Nature.

[10] Huang S., Van Aken O., Schwarzländer M., Belt K., Millar A.H. (2016). The Roles of Mitochondrial Reactive Oxygen Species in Cellular Signaling and Stress Response in Plants. Plant Physiol..

[11] Fang H., Zhou Q., Cheng S., Zhou X., Wei B., Zhao Y., Ji S. (2021). 24-epibrassinolide alleviates postharvest yellowing of broccoli via improving its antioxidant capacity. Food Chem..

[12] Lin Y., Lin H., Zhang S., Chen Y., Chen M., Lin Y. (2014). The role of active oxygen metabolism in hydrogen peroxide-induced pericarp browning of harvested longan fruit. Postharvest Biol. Technol..

[13] Foyer C.H. (2018). Reactive oxygen species, oxidative signaling and the regulation of photosynthesis. Environ. Exp. Bot..

[14] Pandey P., Srivastava R.K., Rajpoot R., Rani A., Pandey A.K., Dubey R.S. (2016). Water deficit and aluminum interactive effects on generation of reactive oxygen species and responses of antioxidative enzymes in the seedlings of two rice cultivars differing in stress tolerance. Environ. Sci. Pollut. Res..

[15] Silva V.M., Rimoldi Tavanti R.F., Gratão P.L., Alcock T.D., dos Reis A.R. (2020). Selenate and selenite affect photosynthetic pigments and ROS scavenging through distinct mechanisms in cowpea (*Vigna unguiculata* (L.) walp) plants. Ecotox. Environ. Saf..

[16] Bai X., Yang Z., Shen W., Shao Y., Zeng J., Li W. (2022). Polyphenol treatment delays the browning of litchi pericarps and promotes the total antioxidant capacity of litchi fruit. Sci. Hortic..

[17] Hasanuzzaman M., Bhuyan M.H.M.B., Anee T.I., Parvin K., Nahar K., Mahmud J.A., Fujita M. (2019). Regulation of Ascorbate-Glutathione Pathway in Mitigating Oxidative Damage in Plants under Abiotic Stress. Antioxidants.

[18] Nadafzadeh M., Abdanan Mehdizadeh S., Soltanikazemi M. (2018). Development of computer vision system to predict peroxidase and polyphenol oxidase enzymes to evaluate the process of banana peel browning using genetic programming modeling. Sci. Hortic..

[19] Yingsanga P., Srilaong V., Kanlayanarat S., Noichinda S., McGlasson W.B. (2008). Relationship between browning and related enzymes (PAL, PPO and POD) in rambutan fruit (*Nephelium lappaceum* Linn.) cvs. Rongrien and See-Chompoo. Postharvest Biol. Technol..

[20] Zhao X., Zhang Y., Ma Y., Zhang L., Jiang Y., Liang H., Wang D. (2021). Inhibitory mechanism of low-oxygen-storage treatment in postharvest internal bluing of radish (*Raphanus sativus*) roots. Food Chem..

[21] Endo H., Miyazaki K., Ose K., Imahori Y. (2019). Hot water treatment to alleviate chilling injury and enhance ascorbate-glutathione cycle in sweet pepper fruit during postharvest cold storage. Sci. Hortic..

[22] Halliwell B. (2006). Reactive Species and Antioxidants. Redox Biology Is a Fundamental Theme of Aerobic Life. Plant Physiol..

[23] Sofo A., Scopa A., Nuzzaci M., Vitti A. (2015). Ascorbate Peroxidase and Catalase Activities and Their Genetic Regulation in Plants Subjected to Drought and Salinity Stresses. Int. J. Mol. Sci..

[24] Anjum N.A., Amreen, Tantray A.Y., Khan N.A., Ahmad A. (2020). Reactive oxygen species detection-approaches in plants: Insights into genetically encoded FRET-based sensors. J. Biotechnol..

[25] Czarnocka W., Karpiński S. (2018). Friend or foe? Reactive oxygen species production, scavenging and signaling in plant response to environmental stresses. Free. Radic. Biol. Med..

[26] Passaia G., Margis-Pinheiro M. (2015). Glutathione peroxidases as redox sensor proteins in plant cells. Plant Sci..

[27] Couto N., Wood J., Barber J. (2016). The role of glutathione reductase and related enzymes on cellular redox homoeostasis network. Free. Radic. Biol. Med..

[28] Kapoor D., Singh S., Kumar V., Romero R., Prasad R., Singh J. (2019). Antioxidant enzymes regulation in plants in reference to reactive oxygen species (ROS) and reactive nitrogen species (RNS). Plant Gene.

[29] Anjum N.A., Gill S.S., Gill R., Hasanuzzaman M., Duarte A.C., Pereira E., Ahmad I., Tuteja R., Tuteja N. (2014). Metal/metalloid stress tolerance in plants: Role of ascorbate, its redox couple, and associated enzymes. Protoplasma.

[30] Asada K. (2006). Production and Scavenging of Reactive Oxygen Species in Chloroplasts and Their Functions. Plant Physiol..

[31] Bhuyan M.H.M.B., Hasanuzzaman M., Mahmud J.A., Hossain M.S., Bhuiyan T.F., Fujita M. (2019). Unraveling Morphophysiological and Biochemical Responses of *Triticum aestivum* L. to Extreme pH: Coordinated Actions of Antioxidant Defense and Glyoxalase Systems. Plants.

[32] Hasanuzzaman M., Nahar K., Rahman A., Mahmud J.A., Alharby H.F., Fujita M. (2018). Exogenous glutathione attenuates lead-induced oxidative stress in wheat by improving antioxidant defense and physiological mechanisms. J. Plant Interact..

[33] Ahmad P., Alam P., Balawi T.H., Altalayan F.H., Ahanger M.A., Ashraf M. (2020). Sodium nitroprusside (SNP) improves tolerance to arsenic (As) toxicity in Vicia faba through the modifications of biochemical attributes, antioxidants, ascorbate-glutathione cycle and glyoxalase cycle. Chemosphere.

[34] de Oliveira F.K., Santos L.O., Buffon J.G. (2021). Mechanism of action, sources, and application of peroxidases. Food Res. Int..

[35] Panadare D., Rathod V.K. (2018). Extraction and purification of polyphenol oxidase: A review. Biocatal. Agric. Biotechnol..

[36] Zhang Y., Zhao X., Ma Y., Jiang Y., Wang D., Liang H. (2021). Comparison of blue discoloration in radish root among different varieties and blue pigment stability analysis. Food Chem..

[37] Cocetta G., Baldassarre V., Spinardi A., Ferrante A. (2014). Effect of cutting on ascorbic acid oxidation and recycling in fresh-cut baby spinach (*Spinacia oleracea* L.) leaves. Postharvest Biol. Technol..

[38] Kuijpers T.F.M., Narváez-Cuenca C.-E., Vincken J.-P., Verloop A.J.W., van Berkel W.J.H., Gruppen H. (2012). Inhibition of Enzymatic Browning of Chlorogenic Acid by Sulfur-Containing Compounds. J. Agric. Food Chem..

[39] Wang J., Wei L., Yan L., Zheng H., Liu C., Zheng L. (2022). Effects of postharvest cysteine treatment on sensory quality and contents of bioactive compounds in goji fruit. Food Chem..

